# Climate change and climate variability: personal motivation for adaptation and mitigation

**DOI:** 10.1186/1476-069X-10-46

**Published:** 2011-05-21

**Authors:** Jan C Semenza, George B Ploubidis, Linda A George

**Affiliations:** 1 Future Threats and Determinants Section, Scientific Advice Unit, European Centre for Disease Prevention and Control (ECDC), Stockholm, Sweden; 2 Centre for Population Studies & Medical Statistics, Unit Department of Epidemiology & Population Health, London School of Hygiene & Tropical Medicine, London, UK; 3 Portland State University, Environmental Sciences and Resources Program, Portland, OR, USA

## Abstract

**Background:**

Global climate change impacts on human and natural systems are predicted to be severe, far reaching, and to affect the most physically and economically vulnerable disproportionately. Society can respond to these threats through two strategies: mitigation and adaptation. Industry, commerce, and government play indispensable roles in these actions but so do individuals, if they are receptive to behavior change. We explored whether the health frame can be used as a context to motivate behavioral reductions of greenhouse gas emissions and adaptation measures.

**Methods:**

In 2008, we conducted a cross-sectional survey in the United States using random digit dialing. Personal relevance of climate change from health threats was explored with the Health Belief Model (HBM) as a conceptual frame and analyzed through logistic regressions and path analysis.

**Results:**

Of 771 individuals surveyed, 81% (n = 622) acknowledged that climate change was occurring, and were aware of the associated ecologic and human health risks. Respondents reported reduced energy consumption if they believed climate change could affect their way of life (perceived susceptibility), Odds Ratio (OR) = 2.4 (95% Confidence Interval (CI): 1.4 - 4.0), endanger their life (perceived severity), OR = 1.9 (95% CI: 1.1 - 3.1), or saw serious barriers to protecting themselves from climate change, OR = 2.1 (95% CI: 1.2 - 3.5). Perceived susceptibility had the strongest effect on reduced energy consumption, either directly or indirectly via perceived severity. Those that reported having the necessary information to prepare for climate change impacts were more likely to have an emergency kit OR = 2.1 (95% CI: 1.4 - 3.1) or plan, OR = 2.2 (95% CI: 1.5 -3.2) for their household, but also saw serious barriers to protecting themselves from climate change or climate variability, either by having an emergency kit OR = 1.6 (95% CI: 1.1 - 2.4) or an emergency plan OR = 1.5 (95%CI: 1.0 - 2.2).

**Conclusions:**

Motivation for voluntary mitigation is mostly dependent on perceived susceptibility to threats and severity of climate change or climate variability impacts, whereas adaptation is largely dependent on the availability of information relevant to climate change. Thus, the climate change discourse could be framed from a health perspective to motivate behaviour change.

## Background

Humans are now unequivocally implicated in contributing to global climate change [1,2]. Strategic action is required both from individuals and the private/public sector to prevent harmful corollaries from climate change to individuals and society at large. Climate change will alter the probability of extreme weather events, which have been associated with adverse health outcomes, such as heat-related mortality and morbidity during heat waves [3,4]; injuries from extreme weather events [5]; injuries and death from flooding [6]; re- and emerging communicable diseases such as hantavirus associated hemorrhagic fever, West Nile fever, or Lyme disease [7]. Dramatic episodes, such as the European heat wave of 2003 or hurricane Katrina in 2005 can be seen in isolation or as part of a probability function of events with increasing frequency, duration, and intensity. Thus, the difficulty in building understanding of climate change lies in the fact that many climate change-related events such as natural disasters or disease outbreaks cannot be directly attributed to climate change making it less intuitive and thus difficult to communicate.

In order to gauge the motivation of individuals to take precautionary steps to reduce climate risks we explored options for mitigation and adaptation. Mitigation entails reductions in greenhouse gas emissions and augmentation in greenhouse gas sinks intended to minimize the extent of global warming [8]. These steps include energy conservation by increasing the fuel efficiency of vehicles; switching to cleaner energy sources by changing business practices; or carbon sequestration through tropical reforestation. However, these practices have proven to be remarkably slow and difficult to implement at best. Even if so, their impact on global climate change will not be noticed in decades to come due to the longevity of greenhouse gases in the atmosphere [2]. Thus, adaptation to climate change impacts becomes a necessity both on an individual and communal level [9,10]. Adaptation entails adjustments of environmental or social settings in response to past, current or anticipated climatic events and their impacts in order to moderate their consequences [2]. Autonomous (or spontaneous) adaptation is typically defined as responding to climate-driven changes in natural systems that occur naturally by private actors without intervention of public institutions [8]. It is usually the result of reactive responses to current climate impacts, rather than preventive measures. In contrast, anticipatory (proactive or planned) adaptation is initiated prior to climate change impacts are observed. It is based on scientific information about projected impacts and is usually executed by government agencies [2,11].

How then can the public be motivated to take mitigation and adaptation steps?

Climate change has traditionally been framed as an environmental, rather than a health issue. Concerns of ecologic, environmental, social, or economic climate change impacts are certainly important drivers of behaviour change but may have contributed to the recent "climate fatigue" [12]. Meanwhile, it is known that personal perception of risk is the strongest motivator of health behaviour change based on the health promotion literature [13]. Potentially then, the health aspects of climate change should resonate well across wide segments of the American public [14]. Thus, health could be a strong motivating factor for individuals to reduce greenhouse gas emissions and to adopt adaptation measures to reduce health risks. However, it is not clear if the health frame would suffice to engage the pubic in adaptation and mitigation steps, since they hinge on public appreciation of the health threats of these climatic processes. Besides, media coverage has been rather polarized and not constructively educated the public about potential health threats [15]. Very little research has been published to date on public perception of adverse health effects from climate change but some studies that have touched on this issue have not found the public to be very knowledgeable [16-18].

The goal of this study was to assess the health context as a motivating factor for adaptation and mitigation behavior. We had previously tested the transtheoretical model (stages of change model) to explain barriers to behaviour change for mitigation [19]. We also considered theory of reasoned action and social learning theory to elucidate underlying drivers of behaviour change [20,21]. In order to test the hypothesis whether health is an appropriate frame for behaviour change in response to climate change risks we applied the health belief model (HBM) to gauge respondents' willingness to engage in voluntary mitigation and adaptation efforts based on their attitudes and beliefs [22]. The HBM had originally been developed to explain the likelihood of health-related behavior from an individual perspective. The HBM has been widely used to understand preventive health behaviors as well as mitigation behavior to reduce environmental pollution that has human health impacts [23-25]. The components of the HBM are perception of susceptibility, severity, benefits, and barriers to action, as well as cues to action and self-efficacy. Perceived susceptibility and perceived barriers have been shown to be associated with preventive behavior while perceived severity, perceived benefits, and perceived barriers have been most strongly associated with treatment of a condition.

We hypothesized that if climate change is perceived as a health threat, then the components of the HBM might be able to predict mitigation and adaptation behavior. We probed whether respondents considered themselves to be susceptible to the threats of climate change/variability and whether this health risk was deemed severe. We explored whether or not the HBM could explain respondents' propensity for autonomous adaptation behavior, and whether or not they had engaged in voluntary energy reduction to counteract global climate change (mitigation behavior). The time-frame for mitigation is long-range while adaptation is more immediate but both actions are important and complementary, and are not mutually exclusive [26]. Thus, these findings are important if public health agencies are to reach the public with behavior change messages through social marketing or communication campaigns using the health context as a frame.

## Methods

### Survey Methods

Attitudes about climate change/variability impacts, mitigation and adaptation were assessed through random-digit dial telephone surveys from a U.S. sample between September and October, 2008 (table 1). The surveys were conducted in both Spanish and English at Portland State University's Survey Research Laboratory (SRL) which has been described elsewhere [19]. The SRL is outfitted with a state-of the-art Computer Assisted Telephone Interviewing (CATI) system with 20 phone interviewing booths. The survey questions appeared on a monitor and were read by the interviewer in a preset order. The survey was designed with complex contingency patterns of questions, where sub-questions were automatically branched off to produce skip patterns. Invalid responses were recognized by the CATI system which enhanced data quality. Furthermore, the need for subsequent data entry was omitted since the data were typed directly into the database. Quality control was assured by a centralized facility that monitored the interviews. The original sample of phone numbers was selected based on the census distribution of population density across all U.S. states in order to assure a geographically representative study population. The optimum time for calling was established through call-back procedures (three call-backs per number) and interview scheduling. On average an interview took 17 minutes and the survey was administered over the course of 33 days. Participants were screened for age (> 18 years), comprehension, zip codes (to assure geographic specificity of respondents) and knowledge of global climate change. Respondents denying climate change as a phenomenon were censured (15%), because all questions pertained to different aspects of climate change [16]. The survey instrument and study protocol was approved by PSU's Human Subjects Research Review Committee (HSRRC Proposal #04157).

**Table 1:** Demographic characteristics of study population, United States, 2008

*Demographic*	*Study Sample N = 622*	*US Sample*	*P Value*
Age (median)	56.0	36.8	p < 0.001*

Gender (female)	56.8%	50.9%	p < 0.05**

Race/Ethnicity			

White	84.9%	75.1%	p < 0.001**

Non-white	13%	24.9%	p < 0.001**

Annual household income			

$30,000 and below	21.2%	29.1%	p < 0.001**

Above $30,000	78.8%	70.9%	p < 0.001**

Highest level of education			

High School diploma or below [Includes GED]	20.9%	44.8%	p < 0.001**

Some College & beyond	79.1%	55.2%	p < 0.001**

*One sample Wilcoxon Signed Ranks test

**Chi Square test

The ranking of respondents per U.S. state in our study population correlated highly with the ranking of the U.S. census population density by state (Spearman correlation: r = 0.897; p < 0.001); thus the study sample was reasonably representative of the geographic distribution of the U.S. population. The demographic profile of the sample (table 1) was also reasonably representative of the US population at large (according to population data from the 2000 census) with respect to gender (Census female 50.9%, analysis sample 56.8%) and race (Census white 75.1%, analysis sample 81.4%), although there was a certain sampling bias towards the age, educational qualifications and household income, since our sample reflected the populations that were more likely to be home during the day, a common occurrence in phone surveys [27-29].

In order to further explain and predict health behaviors related to climate change/variability we drew on the HBM, a psychological model that focuses on attitudes and beliefs of individuals [30]. Survey questions were designed to capture predictors of mitigation and autonomous adaptation behavior through the lens of the HBM components: perception of susceptibility, severity, benefits, and barriers to action, as well as cues to action and self-efficacy. Categorical demographic variables were dichotomized due to sample size constraints (table 1). Responses to open ended questions were subjected to content analysis.

### Statistical modelling

**Figure 1:**
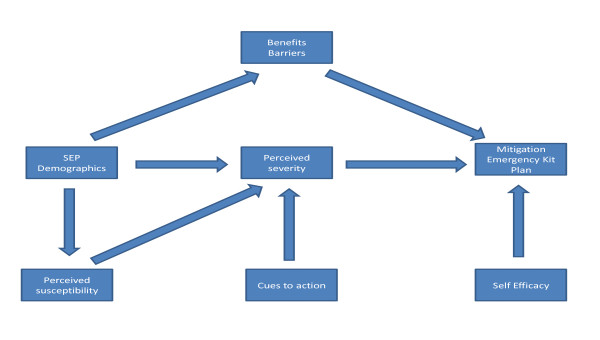
**Conceptual path diagram of the Health Belief Model**.

We first estimated three parallel logistic regression equations within a single model in order to obtain estimates of the association between all the predictors (demographics and HMB related variables) with the three outcomes (mitigation: reduced energy consumption; autonomous adaptation: emergency kit and emergency plan). The causal structure implied by the HBM was not imposed on the data, but the correlation between the three dependent variables was taken into account which were jointly modeled. We employed the robust maximum likelihood (MLR) estimator with Gauss-Hermite integration. In the second stage of the analysis we employed a path analytic approach to estimate the parameters representing the causal structure which is implied by the HBM (see figure 1). For example the effect of perceived susceptibility to the three outcomes is mediated by perceived severity and therefore an indirect effect is implied. Path analysis allows the formal estimation of indirect effects and their associated standard errors, which is not possible with the standard regression approach we used in the first analytic stage, since with this the effect of all the variables in the model is simply adjusted for the presence of all the other covariates [31].

In the presence of binary or ordinal mediators or endogenous variables (perceived severity for example) it has been suggested that the product of standardized probit coefficients is the most reliable estimate of the indirect effect [32]. The probit model assumes that a latent continuum underlies the observed binary or ordinal variables. The standardised probit regression coefficients can therefore be interpreted as the probability of a one standard deviation change in the underlying continuous variable attributable to a one standard deviation change in the predictor. The path analysis was estimated with the Weighted Least Squares, mean and variance adjusted (WLSMV) estimator, in the Mplus 5.21 software [33]. Due to the complexity of the model and the nature of our sample we report significance tests derived by bootstrapped standard errors with 10,000 replications.

## Results

Of the 771 individuals surveyed 3% (n = 23) had never heard of climate change before and 15% (n = 112) did not believe climate change was in fact occurring. However, the majority of Americans (n = 622; 81%) in our survey were aware of climate change and believed that it was certainly taking place. Study participants, when prompted, attributed a number of environmental phenomena to climate change, most noticeably average temperature increases and the melting of permafrost in the Arctic region (table 2). Heat waves, more frequent storms, water shortages, sea-level rise, flooding, and loss of wildlife habitat were mentioned by four out of five respondents that knew about climate change. Misconceptions about climate change impacts were revealed in 10% of comments provided to open-ended questions (n = 135), such as earthquakes, depletion of the ozone layer, vitamin depletion in food, etc. Perceived health risks to the American public are listed in table 3. Respondents were concerned about both air and water quality impacts and about heat stroke and respiratory problems. The most common health concern in the open ended comments related to food shortages (21% of n = 126).

**Table 2:** Perceived environmental, ecological or societal impacts from climate change in the United States, 2008

*Introduction*: I would like to ask you what you think about Global Climate Change. We are conducting a research study about this important issue, which has been discussed in the media a lot. Your opinion about climate change is very important to us. I assure you I am not selling anything. Your experiences will help people understand how to deal with climate change.	
*Screening question*: Have you heard about "global climate change" or "global warming"?	
*Perceived impacts:*	*Proportion*
*Question: Would you say that climate change causes the following types of environmental impacts in the United States?*	
Heat waves (prolonged episodes of hot weather)	0.83
More frequent storms, including hurricanes	0.80
Melting permafrost in the Arctic regions	0.93
Drought conditions or Water shortages	0.84
Forest fires	0.69
Coastal erosion	0.79
Average temperature increase	0.89
Cold waves (blizzards)	0.61
Infectious diseases (e.g. dengue, West Nile Fever, malaria, etc)	0.69
Sea-level rise (gradual)	0.84
Flooding (disaster)	0.80
Aeroallergens (pollen)	0.55
Land or mud slides	0.65
Reduced food production	0.69
Loss of wildlife habitat	0.84
Economic decline	0.51

Note: Restricted to individuals having heard about climate change (N = 622). Not mutually exclusive categories.

**Table 3:** Perceived health risk from climate change, United States, 2008

*Perceived health risk:*	*Proportion*
*Question: Do you think climate change poses a risk to the health of Americans in any of the following ways?*	
Heat stroke or heat exhaustion	0.69

Water quality impacts	0.71

Drowning	0.32

Water-borne diseases	0.59

Infectious diseases (e.g. Dengue, West Nile Fever, Malaria, Pandemic Flu, etc.)	0.61

Air quality impacts	0.82

Respiratory or breathing problems	0.78

Sunburn	0.73

Cancer	0.46

Frostbite or frozen skin	0.32

Stress or anxiety	0.64

Note: Restricted to individuals having heard about climate change (N = 622). Not mutually exclusive categories.

Perceived susceptibility was further explored by asking respondents if climate change could affect their way of life or lifestyle; 78% of respondents recognized a certain level of susceptibility (table 4). Of the respondents, 69% reported that climate change could potentially endanger their lives and pose adverse personal effects. However, 62% were under the impression that personal preparation could save their life. Only a minority of study participants (31%) saw any obstacles or barriers to protecting themselves from negative consequences of climate change. They included lack of money or resources (65%), lack of help from others (56%), lack of knowledge (53%), lack of personal energy or motivation (43%), or lack of time (34%) (table 5). The majority (56%) of respondents felt that they both had the necessary information to prepare for climate change impacts as well as the confidence and ability to protect themselves from health impacts of climate change. Based on these findings we explored whether the respondents were ready for behavioural change and to take mitigation or adaptation actions.

**Table 4:** Survey questions of climate change mitigation and adaptation, United States 2008

*Category*	*Survey Questions*	*Proportion*
Perceived susceptibility	Do you believe climate change could affect your way of life or lifestyle if you don't prepare?	0.78

Perceived severity	Do you believe that climate change can endanger your life?	0.69

Perceived benefits	Can personal preparation for climate change save your life?	0.62

Perceived barriers	Are there serious obstacles and barriers to protecting yourself from negative consequences of climate change?	0.31

Cues to action	Do you think you have the information necessary to prepare for the impacts of climate change?	0.56

Self-efficacy	Do you think that you have the ability and power to protect yourself from dangerous events from climate change?	0.56

Mitigation	Have you reduced your energy consumption in response to what you have heard about global climate change?	0.77

Emergency plan	Does your household currently have a plan for what to do to protect yourself and your family in the event of a disaster or emergency? Such a plan might include how you would evacuate your home, or how to stay in contact with other family members.	0.52

Emergency kit	Some households have an emergency kit that includes such items as a first aid kit, thermometers, flashlight and batteries, food that won't spoil, sufficient drinking water, and other essential things people need to live for at least three days in the event of a disaster or emergency. Does your household have this type of emergency kit?	0.57

Note: Responses were recorded on a binary scale.

**Table 5:** Self-reported obstacles to protect oneself from climate change impacts, United States, 2008

*Serious obstacles and barriers to protecting yourself from negative consequences of climate change:*	*Proportion*
*Question: What are these serious obstacles and barriers to protecting yourself from negative consequences of climate change?*	
You don't know what steps to take to protect yourself	0.53

You lack the skill	0.38

You don't have the personal energy or motivation	0.43

You do not have the time	0.34

You do not have the money or resources	0.65

You lack the help from others	0.56

You feel that it won't make a difference anyway	0.29

You don't believe in climate change	0.10

You believe the government will protect you from climate change	0.10

Other [Please specify]	0.35

Note: Restricted to individuals perceiving serious obstacles and barriers to protecting themselves from negative consequences of climate change (N = 190). Not mutually exclusive categories.

The majority of respondents (77%) reported having reduced their energy consumption based on what they have heard about global climate change. Reported energy conservation steps (N = 479) are listed in table 6. Virtually everybody claimed to have reduced their home energy consumption and their gasoline use. Energy intensive commodities such as water and food were also considered by four out of five respondents. Among those that did not report any energy conservation efforts (18%; n = 118) cited inconvenience and lack of conviction as reasons (table 7).

**Table 6:** Self-reported steps in energy conservation, United Stated, 2008

*Energy conservation strategies:*	*Proportion*
*Question: How did you reduce your energy consumption in response to what you've heard about global climate change?*	
Reduced the amount of gasoline	0.90

Bought a fuel-efficient car	0.44

Started using public transportation, walking, biking or car pooling	0.43

Started recycling	0.82

Reduced your energy consumption at your home	0.99

Reduced your flying	0.49

Bought or switched to renewable energy (power) options	0.32

Conserved water	0.84

Bought locally produced foods	0.81

Reduced meat consumption	0.53

Bought carbon offsets	0.09

Note: Restricted to those that reported reduced energy consumption (N = 479). Not mutually exclusive categories.

**Table 7:** Self-reported obstacles to energy conservation, United States, 2008

*Reason for not reducing energy consumption:*	*Proportion*
*Question: Why have you not reduced your energy consumption in response to global climate change?*	
You do not know what energy consumption to reduce.	0.21
You know what energy consumption to reduce, but you do not know how to change them.	0.30
You do not have the time to reduce your energy consumption.	0.21
You do not have the money to reduce your energy consumption.	0.20
You feel that a reduction in your energy consumption won't make a difference.	0.41
You feel that a reduction in your energy consumption may affect others' opinions of you.	0.08
It is inconvenient to reduce your energy consumption	0.39
You don't believe in global climate change	0.13
You don't believe reducing energy consumption is your responsibility	0.18

Note: Restricted to those that did not reduce their energy consumption (N = 115). Not mutually exclusive categories.

The vast majority of study participants affirmed autonomous adaptive behavior during an extremely hot weather period. These steps included cooling off in a room with air-conditioning (89%) or with a fan (79%), staying out of the sun (96%), drinking plenty of water (99%), and dressing lightly (88%), reduced exercise (60%). For other types of climatic events, 52% individuals reported having an emergency kit that included such items as a first aid kit, thermometers, flashlight and batteries, food that won't spoil, sufficient drinking water, and other essential items in the event of a disaster or emergency. Among those that reported not having one (n = 298) 23% admitted never having thought about it and 3% did not expect to need one; procrastination and laziness were some of the other reasons for not having one. Of those that provided open ended comments (n = 82) 35% reported having some of the items but not all and were planning to reassemble them into a kit in the near future. Qualitative comments described some of the circumstances: "Usually we get tornados, and when there is a tornado there is no time to get the kit when the emergency is happening." and "We are not in a flood plain and once in a while we have had a tornado, but it is so rare. I keep some of the stuff on the shelf but not in a specific kit." Respondents were also asked whether their household currently had a plan for what to do to protect themselves and their family in the event of a disaster or emergency, such as how to evacuate the home, or how to stay in contact with other family members; 57% claimed having such a plan. Of those that did not have an emergency plan (n = 115) 59% never considered it and 5% did not get around to it.

With respect to the predictive power of the HBM constructs as independent variables, in table 8 we present the odd ratios from the three parallel logistic regression models. We observed a positive association between perceived severity and mitigation (OR = 1.874, p < 0.001), perceived susceptibility and mitigation (OR = 2.364, p < 0.001), as well as perceived barriers and mitigation (OR = 2.052, p < 0.001). Furthermore, gender was associated with mitigation (OR = 1.885, p < 0.001), with women being 1.88 times more likely to take voluntary mitigation actions. Having an emergency kit was positively associated with perceived barriers (OR = 1.608, p < 0.001) and cues to action (OR = 2.098, p < 0.001), whereas perceived susceptibility (OR = 1.614, p < 0.001), perceived barriers (OR = 1.476, p < 0.05) and cues to action (OR = 2.161, p < 0.001), were all positively associated with having an emergency plan. Finally gender was negatively associated with having an emergency kit (OR = 0.577, p < 0.001) and an emergency plan (OR = 0.883, p < 0.001), with women being less likely to engage in any of the two autonomous adaptation actions.

**Table 8:** Odds Ratios and 95% Confidence Intervals from the three simultaneous logistic regression models, United States, 2008

	Mitigation	Adaptation - Kit	Adaptation - Plan
Severity	1.874 (1.135 - 3.093)*	1.373 (0.881 - 2.140)	0.817 (0.522 - 1.279)

Susceptibility	2.364 (1.393 - 4.011)**	1.113 (0.680 - 1.823)	1.614 (0.992 - 2.627)

Benefits	1.014 (0.967 - 1.063)	1.015 ( 0.976 - 1.056)	1.002 (0.964 - 1.043)

Barriers	2.052 (1.188 - 3.541)**	1.608 ( 1.080 - 2.394)*	1.476 (1.026 - 2.193)*

Cues to Action	0.866 (0.542 - 1.382)	2.098 (1.430 - 3.078)*	2.161 (1.477 -3.161)*

Self Efficacy	0.672 (0.420 - 1.075)	0.855 (0.585 - 1.249)	1.268 (0.870 - 1.848)

Age	1.002 (0.993 - 1.010)	1.003 (0.989 - 1.012)	1.006 (0.992 - 1.011)

Gender	1.885 (1.204 - 2.951)*	0.577 (0.398 - 0.836)**	0.883 (0.611 - 1.276)*

Income	1.412 (0.770 - 2.590)	0.795 (0.489 - 1.291)	0.982 (0.611 - 1.579)

Education	0.873 (0.480 - 1.588)	1.006 (0.633 - 1.599)	1.077 (0.684 - 1.696)

Housing Tenure	0.489 (0.265 - 0.903)**	0.574 (0.347 - 0.950)**	1.118 (0.671 - 1.861)

Employment	0.959 (0.516 - 1.782)	1.434 (0.841 - 2.446)	0.881 (0.504 - 1.539)

Retired	1.242 (0.630 - 2.450)	1.035 (0.588 - 1.035)	1.688 (0.929 - 3.067)

Race	1.039 (0.548 - 1.970)	1.105 (0.629 - 1.942)	0.877 (0.505 - 1.523)

*p < 0.05

**p < 0.001

In table 9 we present the standardized probit regression coefficients derived from the path analytic model, in order to estimate the influence of each pathway implied by the HBM. We did not observe any significant effect of the demographic characteristics and SEP status of the participants in the endogenous variables -mediators (perceived threat, perceived benefits and perceived barriers) of the HBM. Consequently none of the indirect effects of the demographic and SEP indicators on the three outcomes was significant. On the contrary we observed a strong positive association between perceived severity and mitigation, *β *= 0.479, p < 0.001. Similarly we observed a strong indirect effect of perceived susceptibility on mitigation via perceived severity, *β *= 0.349, p < 0.001, which was dominated by the very strong association between perceived susceptibility and perceived severity, *β *= 0.728, p < 0.001. Perceived benefits had a positive association with mitigation, *β *= 0.204, p < 0.001, as did perceived barriers, *β *= 0.322, p < 0.001. We did not observe any other significant association between the HBM variables and mitigation.

**Table 9:** Standardised probit regression parameters, decomposed to direct and indirect effects, United States, 2008

		Mitigation	Emergency Kit	Emergency Plan	Susceptibility	Severity	Benefits	Barriers
Severity	Direct	0.479**	0.105	0.068				

Susceptibility	Direct					0.728**		

	Indirect via Severity	0.349*	0.100*	0.050				

Benefits	Direct	0.204*	0.108*	0.011				

	Indirect via Severity							

Barriers	Direct	0.322**	0.213*	0.160*				

	Indirect via Severity							

Cues to Action	Direct	-0.018**	0.217	0.227		-0.140*		

	Indirect via Severity	-0.067	-0.023	-0.01				

Self Efficacy	Direct	-0.12	-0.053	0.069				

Age	Direct				0.078	-0.022	-0.036	-0.022

	Indirect Total	0.002	-0.003	-0.002				

	Indirect via Severity	-0.01	-0.004	-0.001				

	Indirect via Benefits	-0.007	-0.004	0				

	Indirect via Barriers	-0.007	-0.005	-0.004				

	Indirect via Susceptibility & Severity	0.027	0.009	0.004				

Gender	Direct	0.146*	-0.165*	-0.101*	0.018	0.063	0.004	-0.016

	Indirect Total	0.032	0.01	0.003				

	Indirect via Severity	0.03	0.01	0.004				

	Indirect via Benefits	0.001	0	0				

	Indirect via Barriers	-0.005	-0.003	-0.003				

	Indirect via Susceptibility & Severity	0.006	0.002	0.001				

Income	Direct				-0.055	-0.11	0.025	-0.011

	Indirect Total	-0.07	-0.024	-0.012				

	Indirect via Severity	-0.053	-0.018	-0.007				

	Indirect via Benefits	0.005	0.003	0				

	Indirect via Barriers	-0.003	-0.002	-0.002				

	Indirect via Suceptibility & Severity	-0.019	-0.007	-0.003				

Education	Direct				0.029	-0.005	-0.127	0.064

	Indirect Total	0.003	0.003	0.01				

	Indirect via Severity	-0.002	-0.001	0				

	Indirect via Benefits	-0.026	-0.014	-0.001				

	Indirect via Barriers	0.021	0.014	0.010				

	Indirect via Suceptibility & Severity	0.01	0.004	0.001				

Housing Tenure	Direct				-0.015	-0.035	0.047	-0.011

	Indirect Total	-0.016	-0.005	-0.004				

	Indirect via Severity	-0.017	-0.006	-0.002				

	Indirect via Benefits	0.01	0.005	0.001				

	Indirect via Barriers	-0.004	-0.002	-0.002				

	Indirect via Suceptibility & Severity	-0.005	-0.002	-0.001				

Employment	Direct				0.028	-0.119	0.164	-0.04

	Indirect Total	-0.027	-0.007	-0.011				

	Indirect via Severity	-0.057	-0.02	-0.008				

	Indirect via Benefits	0.033	0.018	0.002				

	Indirect via Barriers	-0.013	-0.008	-0.006				

	Indirect via Suceptibility & Severity	0.01	0.003	0.001				

Retired	Direct				-0.056	0.16	-0.105	-0.015

	Indirect Total	0.031	0.005	0.005				

	Indirect via Severity	0.077	0.026	0.011				

	Indirect via Benefits	-0.021	-0.011	-0.001				

	Indirect via Barriers	-0.005	-0.003	-0.002				

	Indirect via Suceptibility & Severity	-0.02	-0.007	-0.003				

Race	Direct				0.191	-0.082	0.117	0.021

	Indirect Total	0.093	0.039	0.014				

	Indirect via Severity	-0.039	-0.013	-0.006				

	Indirect via Benefits	0.024	0.013	0.001				

	Indirect via Barriers	0.007	0.005	0.003				

	Indirect via Suceptibility & Severity	0.102	0.035	0.014				

*p < 0.005

** p < 0.001

Underlined parameters are derived from the refined path analysis

With respect to the two autonomous adaptation actions, we observed a positive association between perceived susceptibility and having an emergency kit, *β *= 0.100, p < 0.01, as well as an indirect effect of perceived susceptibility via perceived severity, *β *= 0.108, p < 0.01. Perceived benefits had also a significant association with having an emergency kit, *β *= 0.108, p < 0.01, as did perceived barriers, *β *= 0.213, p < 0.001. We did not observe any other significant association between the HBM variables and having an emergency kit. Finally, perceived barriers had a positive association with having an emergency plan, *β *= 0.160, p < 0.001. We did not observe any other significant associations between the HBM variables and having a plan. Based on the results of both analytic approaches (parallel logistic regressions and path analysis) we estimated a refined path analytic model adding direct effects from cues to action and gender to the three outcomes. The added results are presented as underlined parameters in table 9. As expected from the logistic regression results cues to action were positively associated with having an emergency kit and an emergency plan, whereas gender was positively associated with mitigation and negatively with both autonomous adaptation actions.

## Discussion

In the present study we explored perception of climate change risks among those who were not dismissive of climate change [16]. We tested the predictive power of the HBM with respect to respondents' propensity for autonomous adaptation behavior and mitigation behavior. We employed two statistical modeling approaches in order to test this: i) three parallel logistic regressions to obtain fully adjusted parameters for all the HBM constructs as well as demographics and socio-economic predictors; ii) a path analysis, where the causal structure implied by the HBM (see figure 1) was taken into account in the estimation of the model. This allowed us to estimate the indirect effects of perceived susceptibility, cues to action and demographic characteristics - all via perceived severity - on the three outcomes.

Our findings on environmental impacts from climate change are very similar to recent surveys conducted in the U.S., Canada and Malta with 60-80% of respondents anticipating environmental threats [16]. Climate change or climate variability is perceived as posing a risk which sets the stage for behaviour change. Almost 8 in 10 respondents who had heard about global climate change reported having reduced their energy consumption; this self-reported mitigation effort was associated with a sense of susceptibility as well as the severity of climate change, with the effect of susceptibility being both direct as well as mediated by severity, thus making it the strongest predictor of mitigation effort. Furthermore, while respondents felt that there were certain barriers to protecting themselves from the negative consequences of climate change they also felt that their mitigation actions had co-benefits, such as reduced energy bills.

Intentional reduction in energy consumption by individuals hinges on their state of awareness and concern about climate change, their willingness to act and their ability to change [34,35]. Thus, it is important to portray voluntary mitigation as necessary and achievable [36]. Low impact energy conservation was acted upon but not on high energy savings: curtailment (home energy conservation such as switching off lights; driving less; etc) was readily embraced (or at least reported) in contrast to efficiency improvements (switching to a fuel-efficient car or appliance) which was not, despite the fact, that later would deliver higher energy savings. Other high energy savings activities such as flying less or walking more were comparatively underreported in our survey which has also been reported elsewhere [37]. It is important to note that respondents were prompted about these different activities and might as a result have overstated their true motivation for these actions [38]. While interviewers specifically asked about climate change-related behavior change other factors (such as utility bills and gasoline prices) could also have contributed to behavior change. In addition, we did not attempt to quantify the extent of energy reduction based on these self-reported mitigation activities. These data should be considered an indication that the respondents would be willing to tackle mitigation steps but not necessarily being actively engaged in climate change mitigation.

The majority of respondents also reported having taken steps towards autonomous adaptation to extreme weather events attributable to climate variability or climate change. Study participants that were aware of climate change attributed a number of environmental impacts to climate change such as average temperature increase, heat waves, drought conditions or water shortage. Heat stroke or heat exhaustion, stress or anxieties were listed as major health concerns from these environmental impacts. In the context of the climate change interview, over half of respondents (52%) asserted having prepared an emergency kit with essential items needed in the event of a disaster or emergency and 57% claimed having a household emergency plan to protect themselves and their family in the event of a disaster or emergency. Both of these autonomous adaptation actions were positively influenced by cues to action, a finding which indicates that respondents felt they had the necessary information to prepare for the impacts of climate change. Similarly, both autonomous adaptation actions were influenced by perceived barriers, indicating that without removing those barriers the necessary information (cues to action) may not result in the desired behavioural change which is needed for successful adaptation. The majority of these barriers could be overcome with financial or practical support. There are a number of other autonomous adaptation actions or reactive responses to current climate impacts not covered in our survey. They could be supported with tax incentives and technical solutions and government agencies should work with communities to address their needs. From the other predictors only gender was associated with either autonomous adaptation action, with women appearing to be less adaptive. The majority of study participants had adapted to extreme weather conditions by cooling off in air conditioned places, reducing physical exertion or using a fan. These autonomous adaptation actions are reactive in nature, more so than proactive and thus does not capture anticipatory (proactive or planned) adaptive intentions. The survey instrument was initially designed to capture anticipatory adaptation as well by asking respondents if they adapted their home to climate change. For example: have you installed an A/C, insulation, insect screens, eliminated mosquitoes breeding sites, etc. However, due to low frequency responses these questions were eliminated from the analysis; thus the results apply to autonomous adaptation only and not to long-term impacts. Other studies that did not specifically examine the psychological constructs of the HBM have shown that adaptive behaviour to climate change may be more strongly linked to factors such as, environmental attitudes, political affiliation and attitudes towards scientists [39-41]. The relative differences in predictors of our mitigation and adaptation outcomes might in part be due to the wording of the survey questions. Nevertheless, health as a communication frame can be used to complement other strategies to augment the public response [42].

Attitudes and public perception of global climate change has also been examined in other surveys in the U.S. [19,23,40,41,43-47]. A recent study of US local public health department directors found that health directors are not actively responding to climate change in part due to their belief that the public does not have knowledge about the impact of climate change and therefore would be unwilling to support mitigation and adaptation activities [48]. Our study indicates that the majority of the public report awareness of environmental and health risks associated with climate change and that they consider themselves to be susceptible to being affected by it. Individuals with concerns about climate change hazards have been shown to be more engaged in personal actions [49,50]. Our survey examined more specifically vulnerability and risk perception of climate change/vulnerability and indicates that the majority of the respondents would like to be part of climate solutions, which is consistent with other studies [51-54].

## Conclusions

These findings indicate that the motivation for voluntary mitigation is mostly dependent on the perceived susceptibility to and severity of climate change, and autonomous adaptation is largely dependent on the availability of information relevant to climate change and its impact. Furthermore, our findings suggest an extension of the classic HBM as a predictive causal structure of mitigation and autonomous adaptation strategies, by adding direct effects of cues to action and gender to the these outcomes. Media advocacy campaigns should embrace the health context as a frame and aim at increasing general understanding of climate change and encourage active participation in mitigation and adaptation. Our findings indicate that proximal climate threats against which individuals feel highly susceptible, such as heat waves, droughts, or forest fires, are acted upon, especially when having the necessary information and if the threat is perceived as endangering their way of life. However, climate change is a multiplier of existing vulnerabilities for susceptible populations, underrepresented in our survey, who are at increased risk from such events. Vulnerable populations of low socio-economic status tend not to respond equally well to health promotion campaigns compared to the general population [55]. Thus, traditional media messages might not be able to persuade these populations to change behaviour and concerted efforts need to be put in place to reach these individuals both through more effective communication frames and community organizing [10,56]. This study indicates how climate change can be framed from a health perspective to advance population health.

## List of Abbreviations

OR: Odds Ratio; CI: Confidence Interval; HBM: Health Belief Model; SRL: Survey Research Laboratory; CATI: Computer Assisted Telephone Interviewing; HSRRC: Human Subjects Research Review Committee; A/C: Air Conditioning.

## Competing interests

The authors declare that they have no competing interests.

## Authors' contributions

JCS designed the study, developed the questionnaire, supervised the survey, analyzed the data and wrote the paper. GBP conducted the parallel logistic regressions, path analysis and contributed to the writing. LAG contributed to the questionnaire development, survey, and writing of the paper. All authors read and approved the final manuscript
